# Familial idiopathic normal pressure hydrocephalus

**DOI:** 10.1186/2045-8118-12-S1-O43

**Published:** 2015-09-18

**Authors:** Joel Huovinen, Sami Kastinen, Simo Komulainen, Minna Oinas, Cecilia Avellan, Janek Franzen, Jaakko Rinne, Antti Ronkainen, Mikko Kauppinen, Kimmo Lönroth, Markus Perola, Okko T Pyykkö, Anne M Koivisto, Anne M Remes, Mikko Hiltunen, Seppo Helisalmi, Mitja Kurki, Juha E Jääskeläinen, Ville Leinonen

**Affiliations:** 1 Department of Neurosurgery, Kuopio University Hospital and Neurosurgery, Institute of Clinical Medicine, University of Eastern Finland, Kuopio, Finland; 2 Department of Neurosurgery, University of Helsinki and Helsinki University Hospital, Finland; 3 Clinical Neurosciences, Department of Neurosurgery, Turku University Hospital, Turku, Finland; 4 Department of Neurosurgery, Tampere University Hospital, Tampere, Finland; 5 Department of Neurosurgery, Oulu University Hospital, Oulu, Finland; 6 South Ostrobothnia Central Hospital, Seinäjoki, Finland; 7 National Institute for Health and Welfare, Finland and University of Helsinki, Helsinki, Finland; 8 Unit of Neurology, Institute of Clinical Medicine, University of Eastern Finland, Kuopio, Finland and Department of Neurology, Kuopio University Hospital, Kuopio, Finland; 9 Institute of Biomedicine, University of Eastern Finland, Kuopio, Finland; 10 Analytical and Translational Genetics Unit, Department of Medicine, Massachusetts General Hospital, USA, Program in Medical and Population Genetics, Broad Institute of MIT and Harvard, USA Stanley Center for Psychiatric Research

## Introduction

Idiopathic normal pressure hydrocephalus (iNPH) is a late-onset, surgically alleviated, progressive neurodegenerative disease with unknown etiology. There are few studies describing pedigrees with multiple affected relatives. Our aim was to identify and characterize a potential familial subgroup of idiopathic normal pressure hydrocephalus in a nation-wide Finnish cohort

## Methods

Overall 375 iNPH-patients operated between 1993 and 2014 were questionnaired and phone interviewed, whether they have relatives with either diagnosed iNPH or disease-related symptomatology. Genograms of families with such findings were drawn.

## Results

60 patients (16 %) had potential familial iNPH. 18 patients from 12 separate pedigrees had at least one relative shunted due to iNPH. Patients with familial iNPH reported a complete triad of NPH-symptoms (p=0.03) and memory problems (p=0.014) more often than sporadic cases. Both shunted and symptomatic relatives were mainly first-degree.

According to age-adjusted multivariate logistic regression analysis diagnosed dementia (odds ratio [OR] 2.9; 95% confidence interval [CI], 1.5–5.4) and nonarthritic rheumatoid etiologies (OR, 4.4; 95% CI, 1.6–11.7) were more frequent in familial than sporadic patients. Geographical variation in the occurrence of iNPH was observed, the incidence being highest in Eastern Finland. Frequency of APOE epsilon 4 as well as diagnosed Alzheimer's disease (AD) and AD-medication were similar in familial and sporadic iNPH-patients.

## Conclusions

This study indicates a familial entity of iNPH offering a novel approach to discover the potential genetic characteristics of iNPH. Furthermore, these pedigrees offer an intriguing opportunity to conduct longitudinal studies focusing on potential preclinical signs of iNPH. Our findings support iNPH as a specific neurodegenerative disease.

